# Release efficiencies of potassium permanganate controlled-release biodegradable polymer (CRBP) pellets embedded in polyvinyl acetate (CRBP-PVAc) and polyethylene oxide (CRBP- PEO) for groundwater treatment

**DOI:** 10.1016/j.heliyon.2023.e20858

**Published:** 2023-10-10

**Authors:** Mehdi Lamssali, Stephanie Luster-Teasley, Dongyang Deng, Nafisa Sirelkhatim, Yen Doan, Mosarrat Samiha Kabir, Qingan Zeng

**Affiliations:** a Department of Built Environment, North Carolina A&T State University, 27411, Greensboro, NC, United States; b Provost and VC for Academic Affairs, North Carolina A&T State University, 27411, Greensboro, NC, United States; c Dean's Office, Joint School of Nanoscience and Nanoengineering, 27401, Greensboro, NC, United States; d Department of Civil, Architectural and Environmental Engineering, North Carolina A&T State University, 27411, Greensboro, NC, United States; e Department of Nanoengineering, Joint School of Nanoscience and Nanoengineering, 27401, Greensboro, NC, United States; f Department of Computer Systems Technology, North Carolina A&T State University, 27411, Greensboro, NC, United States

**Keywords:** Controlled-release, Potassium permanganate, Encapsulation, Polyvinyl acetate, Polyethylene oxide, In-situ chemical oxidation, Groundwater treatment

## Abstract

In-situ chemical oxidation (ISCO) is a commonly used method for the remediation of environmental contaminants in groundwater systems. However, traditional ISCO methods are associated with several limitations, including safety and handling concerns, rebound of groundwater contaminants, and difficulty in reaching all areas of contamination. To overcome these limitations, novel Controlled-Release Biodegradable Polymer (CRBP) pellets containing the oxidant KMnO₄ were designed and tested. The CRBP pellets were encapsulated in Polyvinyl Acetate (CRBP-PVAc) and Polyethylene Oxide (CRBP-PEO) at different weight percentages, baking temperatures, and time. Their release efficiency was tested in water, soil, and water and soil mixture media. Results showed that CRBP-PVAc pellets with 60 % KMnO₄ and baked at 120 °C for 2 min had the highest release percentage and rate across different conditions tested. Natural organic matter was also found to be an important factor to consider for in-field applications due to its potential reducing effect with $\text{Mn}O_{4}^{-}$. Overall, the use of CRBP pellets offers an innovative and sustainable solution to remediate contaminated groundwater systems, with the potential to overcome traditional ISCO limitations. These findings suggest that CRBP pellets could provide sustained and controlled release of the oxidant, reducing the need for multiple injections and minimizing safety and handling concerns. This study represents an important step towards developing a new and effective approach for ISCO remediation.

## Highlights

• CRBP pellets offer an effective treatment for groundwater contaminants.
• CRBP pellets reduce the need of multiple injections and safety and handling risks.
• CRBP-PVAc (60 % KMnO_4_ and 120 °C for 2 min) is found to be the most effective.

## Graphical abstract

**Figure:**
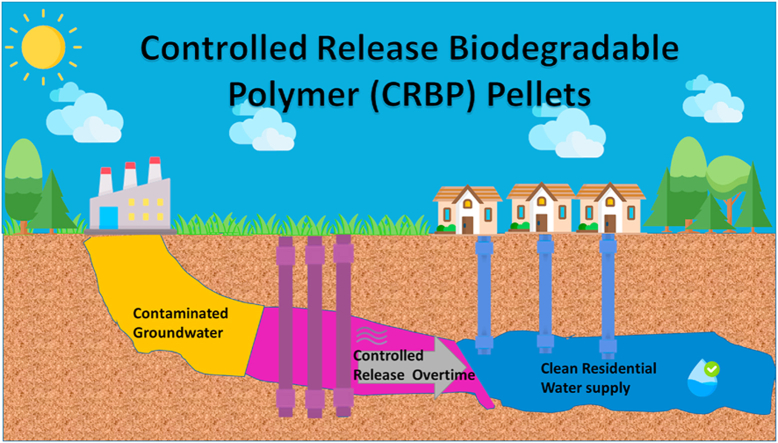

## 1 Introduction

Potassium Permanganate (KMnO₄) has been widely used as a strong oxidant for the remediation of groundwater contaminated by chlorinated ethylenes such as trichloroethylene (TCE), perchloroethylene (PCE), Vinyl Chloride (VC), and dichloroethylene (DCE) [[1], [2], [3], [4]] due to its relatively high stability, oxidation potential, diffusion in low permeability zones and cost-efficiency [5]. Studies have demonstrated that KMnO₄ can oxidize chlorinated ethylene compounds into both easily degradable and non-hazardous by-products such as hydrogen chloride, carbon dioxide, and organic acids through spontaneous cleavage of carbon-carbon bond [1,[5], [6], [7], [8], [9]]. Additionally, KMnO₄ has also been reported to degrade phenolic materials into quinone and other compounds as a result of ring cleavage [10]. While KMnO₄ has shown high effectiveness in treating persistent organic compounds from the above-mentioned studies, there are literatures focusing on health-related risks associated with handling KMnO₄, such as workers’ exposure during oxidant injection, explosion due to accidental mixing with combustible or flammable materials, and self-accelerated decomposition if the oxidant is exposed to moisture or heat [9,11,12].

Groundwater remediation undertakes the cleaning and treatment of groundwater that has been contaminated by man-made byproducts such as gasoline, pesticides, and other persistent chemicals [13]. It generally involves the use of various methodologies, which includes: air sparging, monitored natural attenuation, *in-situ* chemical oxidation, controlled release technology, phytoremediation, electrochemical reduction, and enhanced reductive dechlorination, etc. [8]. Air sparging involves the injection of gas (oxygen/air) below the water table in order to remediate contaminated groundwater [14]. The role of the injected oxygen is to volatilize the contaminants dissolved in the groundwater into their vapor phase allowing them to migrate upwards to the vadose zone where they can be extracted [8,15]. Its main advantages are low cost and easy installation but it has limited application in low permeability zones and deep aquifers [8,16]. Monitored natural attenuation, on the other hand, reduces contaminants by naturally occurring subsurface processes (sorption, dilution, volatilization, and biodegradation [17,18] in both groundwater and soil [9]. Its major advantages include low cost, non-intrusiveness, and high affinity with other technologies such as ozone treatment, bioremediation, or phytoremediation but also has limitations of relatively slow removal rates, gradual accumulation of hazardous metabolites, and the need for extensive evaluation and long-term monitoring [8,[19], [20], [21], [22]]. Alternatively, *In-situ* Chemical Oxidation (ISCO) has been majorly used due to its potential in degrading a wide range of organic contaminants such as Polychlorinated Biphenyls (PCBs), Polycyclic Aromatic Hydrocarbons (PAHs), Volatile Organic Compounds (VOCs), and Benzene, Toluene, Ethylbenzene and Xylene (BTEX) into less potent byproducts. This remediation is done using a variety of oxidants which include KMnO₄, hydrogen peroxide (H_2_O_2_), and sodium persulfate (Na_2_S_2_O_8_) [9]. Studies have also reported using ISCO methods in conjunction with other treatment methods such as thermal remediation, air sparging, or bioremediation [[23], [24], [25]]. The combined use of remediation technologies with ISCO has been assessed to enhance the effectiveness of site cleanup by reducing oxidant usage, decreasing treatment time, minimizing treatment costs, and refining residual contaminant removal through post-treatment practices [9,[26], [27], [28]]. Some of the main advantages of ISCO include the ability to use a variety of oxidants, the potential to integrate with multiple delivery approaches, and an aptitude for quick implementation [9,29]. However, due to contaminant rebound, multiple direct injections of oxidants such as KMnO₄ into the groundwater wells is necessary resulting in lower efficiency [30]. Other limitations of ISCO include back diffusion, reduced treatment efficiency of contaminants, and tailing [9,31,32].

Studies have also looked into the application of controlled release technology as an alternative to the traditional ISCO methods for delivering oxidants to gradually release the chemical in a controlled manner. Researchers investigated the release of pellets where KMnO₄ was encapsulated in a paraffin wax matrix [33]. Results suggested a biphasic release with an observed initial rapid diffusion (<10 min) and then followed by a more sustained and slower spread at later stages. KMnO₄ was also encapsulated in Polymethyl Methacrylate (PMMA) glassy polymer-controlled release pellets and demonstrated a release percentage of 79 %, 55 %, and 33 % for the mass ratio of KMnO₄ to PMMA 2:1, 4:1 and 8:1 respectively in a 10-h period [34]. Researchers also investigated the treatment of TCE in groundwater using KMnO₄ encapsulated in slow-release paraffin wax candles [35]. Experimental results showed a 10 %–66 % of TCE removal during a controlled release period of 15 months. Another innovative controlled release technology was also developed by encapsulating KMnO₄ into biodegradable polymers which were both hydrophobic and hydrophilic in nature [[36], [37], [38]]. The polymer pellets were able to gradually release KMnO₄ over a relatively long period of time (44–47 days).

All the above-mentioned technologies have shown high potential in groundwater remediation. However, no studies have developed Controlled-Release Biodegradable Polymer (CRBP) pellets using Polyvinyl Acetate (PVAc) and Polyethylene Oxide (PEO) as the polymer base for KMnO₄. These polymers are used both in the drug and food industry sectors and their applications include controlled drug release for wound healing and sustainable pesticide usage to maintain food security, respectively [39,40]. Some of the main advantages of using these polymers are high biodegradability and efficiency when it comes to the retardation of the dissolution of drugs and chemicals [41]. Other advantages include a very low melting point (60 °C), excellent membrane-forming capability, non-toxicity for environmental applications, and remarkable mixing compatibility with other materials [42,43].

PVAc and PEO are two commonly used polymers with notable biodegradable properties. The biodegradation of PVAc occurs primarily through the action of microorganisms present in the soil or water, which break down the polymer into smaller, non-toxic compounds [44,45]. This is a two-step process that involves the initial hydrolysis of the acetate groups present in the PVAc, followed by the biodegradation of the resulting polyvinyl alcohol (PVA) by microorganisms [[46], [47], [48]]. Specific enzymes produced by microorganisms, such as esterases and lipases, catalyze the hydrolysis of the acetate groups in PVAc [[49], [50], [51]]. This reaction can be represented as follows:

(1) $$PVAC+H_{2}O\underset{esterases/lipases}{\to }PVA+CH3COOH$$

Once the acetate groups are hydrolyzed, the resulting PVA is further biodegraded by microorganisms into carbon dioxide and water [52].

(2) $$PVA+O_{2}\underset{microorganisms}{\to }CO_{2}+H_{2}O$$

On the other hand, PEO undergoes enzymatic degradation or hydrolytic degradation, breaking down into smaller compounds that can be further metabolized by microorganisms [53,54]. The enzymatic degradation of PEO involves the action of enzymes, such as oxidases and peroxidases, which break down the polymer into smaller compounds, including ethylene glycol and oligomers of ethylene oxide [[55], [56], [57], [58]]. The general reaction can be represented as:

(3) $$PEO+H_{2}O\underset{oxidases/peroxidases}{\to }Ethyl\;Glycol+Ethylene\;Oxide\;Oligomers$$

These smaller compounds can then be further metabolized by microorganisms into carbon dioxide and water [57,59]:

(4) $$Ethylene\;Glycol+O2\underset{microorganisms}{\to }CO_{2}+H_{2}O$$

The use of biodegradable polymers like PVAc and PEO in environmental applications has broader implications for sustainable groundwater remediation [57,58,60]. The complete degradation of these polymers into environmentally benign compounds such as carbon dioxide and water ensure that no harmful residues are left in the environment post-treatment [59]. By utilizing biodegradable polymers that break down into non-toxic compounds, the environmental impact of the remediation process is minimized.

Consequently, PVAc and PEO polymers were considered optimum coating materials for developing CRBP pellets. Additionally, CRBP is an innovative ISCO method that can minimize back diffusion, tailing, and persistent plume issues while protecting workers.

In this study, we developed CRBP pellets that use PVAc and PEO as base polymers and KMnO₄ as the oxidant for groundwater applications. The purposes of this study are to:

● Develop the optimum recipe of Controlled-Release Biodegradable Polymer (CRBP) pellets for groundwater treatment based on different polymer types, baking time, baking temperature, and KMnO₄/polymer mass ratios.
● Investigate and compare the release kinetics of CRBP pellets using PVAc and PEO respectively in water, soil and water + soil mixture media.

## 2 Materials and methods

### 2.1 Chemicals and soils

Certified analytical grade KMnO₄ (Certified ACS, 99 %), PVAc, PEO were purchased from Fisher Scientific. DI water was obtained using an ultrapure water system (Milli-Q® Direct Water Purification System).

Pre-sieved silica sand was obtained from AxNano company, whereas clay was collected from NC A&T State University Farm at the location shown in Fig. S1 (36°04'.94″N, 79°44′02″W, max elevation: 783 ft, slope: 0.2 %). Silt was obtained near the city of Asheboro at the location presented in Fig. S2 (35°35′34″N, 79°48′20″W, max elevation: 767 ft, slope: 0.2%). Clay was sieved to obtain particles that are smaller than 0.002 inches in diameter. Silt was sieved to acquire particles between 0.002 and 0.005 inches in diameter. The porosity (n) of the sand, silt, and clay was experimentally determined using the water displacement method and calculated using the formula:

(5) $$n=\frac{V_{v}}{V_{t}}$$

Where $V_{v}$ is the volume of voids, determined by the volume of water added to saturate the soil in a 100 ml graduated cylinder, and $V_{t}$ is the total volume of the soil sample. Measurements were made in triplicate. The average porosity values for sand, silt, and clay were 0.5, 0.55, and 0.62, respectively.

### 2.2 CRBP encapsulation protocol

Controlled Release Biodegradable Polymer pellets were generated using a pellet press acquired from Parr Company (Model 2811 Pellet Press). The pellet press was used to form the pellets by encapsulating KMnO₄ within the PVAc and PEO and letting them cook at varying temperatures and time. Firstly, PVAc and PEO polymers were heated to melt at 60 and 70 °C respectively, and then crystalline KMnO₄ was added. The KMnO₄ particles were uniformly dispersed inside the polymer shell matrix using a stainless-steel spatula to form a relatively homogeneous mixture and then pressed using the pellet press. Afterward, the final mass ratios of KMnO₄ to the CRBP pellet obtained were 20 wt%, 40 wt%, and 60 wt% respectively, and they had an average total mass of 0.3 g, with a diameter of 1 inch. The produced pellets were baked at various temperatures (100 °C, 120 °C, and 140 °C) in combination with varying baking time (2 min, 4 min, and 10 min) and were left to cool down till forming a solidified CRBP structure (Table S1). Fig. S3 demonstrates CRBP pellet pictures before and after treatment, while Fig. S4 represents a schematic diagram depicting the CRBP pellets formation process. Table S2 summarizes the composition of both CRBP-PEO and CRBP-PVAc pellets.

### 2.3 CRBP release experiments

#### 2.3.1 Release in water

In order to determine the release rate of CRBP pellets in water, batch experimental tests were conducted. Direct injections of KMnO_4_ in water serves as negative control and it was observed that it takes around 10 s for KMnO_4_ to fully dissolve in water. The CRBP pellets were added to 500 ml of DI water and were continuously mixed. 10 ml samples were collected at the specific time intervals of 0.5, 1, 5, 10, 15, 20, 60, 120, and 180 min. Triplicate measurements were performed. The KMnO₄ concentration of the collected samples was measured using a Hach DR-3900 spectrophotometer (wavelength of 525 nm). Release studies were performed using two rounds of experiments, round 1, was a comparison between the performance of CRBP-PEO and CRBP-PVAc pellets both containing 20 % and 40 % KMnO₄ and baked at 100 °C for 10 min, respectively. Then, CRBP-PEO pellets were discarded due to their lower release potential compared to CRBP-PVAC pellets. After comparing the two pellets (CRBP-PEO and CRBP-PVAc), round 2 experiments for kinetic studies of CRBP pellets containing 20 %, 40 %, and 60 % KMnO₄ encapsulated in PVAc (baked at 120 °C and 140 °C for 2, 4, and 10 min respectively) were completed.

#### 2.3.2 Release in soil

In order to determine the release rates of CRBP in soil, soil column tests were performed. Based on the water test results, the CRBP-PVAc pellets containing 60 % KMnO₄ and baked at 120 °C for 2 min were chosen as the optimum recipe for the controlled release experiments in soil.

Glass columns (Kimble Kontes Chromaflex) (Inner Diameter: 2.5 cm, length: 15 cm, volume: 73.63 ml) were used for soil column testing. Fig. S5 presents the experimental setup. Soil compaction was performed using a shaker attached to the soil column. For CRBP-PVAc pellets release experiments in sand, soil columns were composed of 100% sand. For CRBP pellet release experiments in clay, soil columns were composed of 50% sand and 50% clay. Finally, for CRBP release experiments in silt, soil columns were composed of 50% sand and 50 % silt. The mixture of sand with silt or clay soil column was used because of the low porosity of clay and silt and to prevent water overflow due to pressure build-up. 1 L Buffer solutions (PBS buffer 10x: 17.8 g of Na2HPO4, 2.4 g of KH2PO4, 80 g of NaCl, 2 g of KCl, and 100 ml of DI water) were used to rinse the soil columns and prepare the columns as pH 6 or 8 using hydrochloric acid. Then once the columns were prepared, the buffer solutions were pumped through the soil column containing CRBP pellets. A constant flow rate (Q = 5 ml/min) was maintained using the peristaltic pump (Cole-Parmer Masterflex pump model 7553–00). 10 ml of samples were then continuously collected every 2 min and KMnO_4_ was measured until a very low absorbance was obtained. Triplicate measurements were performed. The longevity of the CRBP pellets was determined through calculating the number of pore volumes in each experimental run and comparing it to the release efficiency. The number of pore volumes for each soil column were calculate using the following equation:

(6) $$Number\;of\;Pore\;Volumes=\frac{Total\;Volume\;of\;Water\;Added}{Volume\;of\;the\;Soil\;Column\;\left(ml\right)\times Porosity\;of\;Sand}$$

#### 2.3.3 Release in a mixture of soil and water

Lastly, batch tests were also performed by mixing soil with water in order to study the presence of Natural Organic Matter (NOM) and investigate if there is any significant consumption of KMnO_4_ by NOM. Briefly, CRBP-PVAc pellets containing 60% KMnO₄ and baked at 120 °C for 2 min were added to beakers containing DI water and soil (sand/silt/clay). For release experiments in the sand and water, 115 g of sand was used. For the release experiments in silt, the mass of silt and water, 44 g of silt and 44 g of sand were used. For the release experiments in clay, 30 g of clay and 30 g of sand were used. The volume of DI water used was 400 ml 10 ml samples were collected at specific time intervals of 0.5, 1, 5, 10, 15, 20, 60, 120, and 180 min. A Hach DR-3900 spectrophotometer (wavelength of 525 nm) was used to measure the KMnO₄ concentration of the collected samples.

### 2.4 KMnO₄ release and model calculations

The Hach DR3900 spectrophotometer was used to determine the KMnO₄ concentration in the collected samples. This was done by converting the obtained absorbance to a KMnO₄ concentration value by using the following calibration equation:

(7) $$\begin{array}{c} Concentration\;\left(\frac{mg}{l}\right)=\frac{Abs-0.0488}{0.0385} \end{array}$$

KMnO₄ release percentage was calculated using the following equation:

(8) $$\begin{array}{c} KMnO_{4}\;release\;percentage=\frac{Mass\;of\;KMnO_{4}\;released\;\left(mg\right)}{Initial\;mass\;of\;KmnO4\;in\;the\;pellet\;\left(mg\right)}*100\\ \# \end{array}$$

In order to understand the release mechanisms behind the CRBP pellets, multiple pellet release models were investigated to fit with the experimental data obtained. Results were observed to best fit the Kosemeyer-Peppas model ($r^{2}>0.90)$ as opposed to the zero-order and Higuchi models ($r^{2}<0.90$) and was thus used as a model of choice for the CRBP release results. The Kosemeyer-Peppas model is useful when the release mechanism is unknown or when more than one type of drug/oxidant release phenomenon is involved. This model is expressed best by the following Kosemeyer-Peppas equation [61]:

(9) $$\begin{array}{c} Q=Kt^{n} \end{array}$$

where *Q* is the mass fraction of the oxidant at time *t*, *K* is the rate constant ($t^{-n}$) and n is the diffusional exponent. Equation (9) can then be further linearized into the following logarithmic form:

(10) $$\begin{array}{c} \log\;Q_{t}=logK+nlogt \end{array}$$

that can be used to generate values of *n* and *k*. More specifically, the DDsolver software tool was used to generate these model output results [62].

### 2.5 Statistical analysis

In order to determine if there are any significant changes in oxidant release related to variations in pH within the soil release experiments, a one-way analysis also known as ANOVA was performed using the software IBM SPSS Statistics 25 to test the validity of hypothesis testing described as follows:

(11) $$\begin{array}{c} H_{0}:\mu 1=\mu 2=\cdots \end{array}$$

(12) $$\begin{array}{c} H_{a}:\mu 1\neq \mu 2\neq \cdots \end{array}$$

This test is considered an extension of the standardized *t*-test and aims to determine if there exists a considerable statistical difference between the means of two or more independent groups. Obtained p-values were then compared to significance level ***α*** = 0.05. Obtained p-value >0.05 suggested that there is enough evidence to fail to reject the null hypothesis $H_{0}$ while a p-value<0.05 suggested that there is enough evidence to reject the null hypothesis $H_{0}$.

## 3 Results and discussion

### 3.1 Release in water

#### 3.1.1 CRBP-PEO & CRBP-PVAc pellet studies

##### 3.1.1.1 Kinetic release of 20 % and 40 % of KMnO₄ in CRBP-PEO and CRBP-PVAc pellets cooked for 10 min at 100 °C in water

**Fig. 1:**
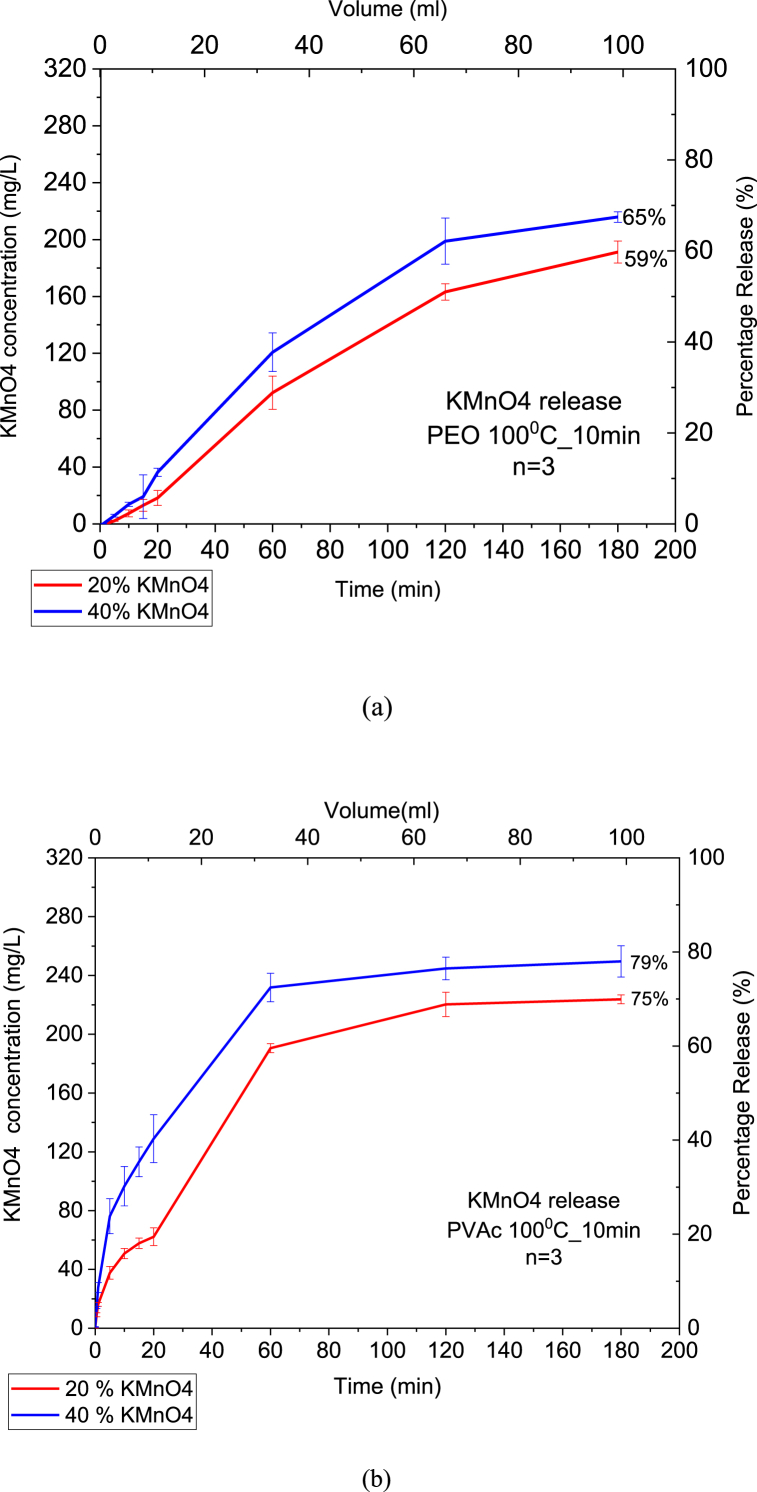
KMnO₄ percentage and concentration release in water for CRBP-PEO (a) and CRBP-PVAc pellets (b) containing 20 % and 40 % KMnO₄ cooked for 100 °C at 10 min.

KMnO₄ release into groundwater was the main focus of this study in order to investigate both the CRBP-PEO and CRBP-PVAc release efficiency of formed pellets. Fig. 1 demonstrates the release profiles over a 3-h period obtained for both CRBP-PEO and CRBP-PVAc containing 20 % and 40 % of KMnO₄ and baked at 100 °C for 10 min, respectively. The lowest release percentage (59 %) was observed with CRBP-PEO pellets containing 20 % of KMnO₄ while the CRBP-PEO pellets with 40 % of KMnO₄ had a release percentage of 65 % (Fig. 1a, Table S3). Comparatively, 20 % and 40 % of KMnO₄ CRBP-PVAc pellets yielded an average release of 75 % and 80 % respectively (Fig. 1b, Table S4). The results of this study indicate that as the mass ratio of KMnO₄ to polymer in the matrix increases, the release percentage of KMnO₄ also increases for both PVAc and PEO polymers, when the mass ratio increases from 0.25:1 to 0.67:1. Similar findings were reported when encapsulating KMnO₄ in a Poly (methyl methacrylate) (PMMA) polymer bead matrix [34]. The study demonstrated that as the polymer content within the release pellet increases, the percentage of KMnO₄ release decreases, this is evident when comparing the release percentages at the 10-h mark, 79 % for 1:2, 55 % for 1:4, and 33 % for 1:8 KMnO₄: polymer mass ratios respectively [34]. This is because as KMnO₄ is depleted at the pellet surfaces, crevices or empty pores will be left in the CRBP, allowing more water to penetrate the polymer matrix and further dissolve the encapsulated oxidant, especially for soluble oxidants such as KMnO₄ with a solubility of 5.0 g/100 ml in water [41]. Thus, increasing the mass ratio of the oxidant KMnO₄ in the CRBP pellet will result in higher release percentages of the oxidant within the pellet. This is also proven by kinetic release experiments of CRBP-PVAc pellets in soil medium, discussed in the later sections.

The experimental data also suggests that the dissolution of KMnO₄ from CRBP-PVAc and CRBP-PEO pellets was observed to follow a biphasic behavior with an initial rapid release accompanied by a slower and more sustained release of KMnO₄ afterward. For CRBP-PVAc pellets, the most rapid release occurred during the initial experiment time (<20 min) compared to CRBP-PEO pellets where the release occurred at a later delayed period (20–40 min). Biphasic release behavior was also observed for other water-soluble oxidants such as Ferrate (VI) and sodium persulfate as reported in previous studies [[63], [64], [65], [66]]. Another research study observed a similar biphasic release behavior when generating Encapsulated Potassium Permanganate (EPP) pellets using wax with 20 % KMnO₄ where early oxidant release was observed (<10 min) due to dissolution of KMnO₄ on the surface or partially embedded in the wax, then later was observed to follow a more sustained release at later stages [67]. This release trend can be explained by the inward occurrence of secondary permeability from outside the polymer matrix through the dissolution-diffusion release mechanism of KMnO₄ readily accessible to water [[68], [69], [70]]. This is especially relevant for polymeric pellets following a Fickian diffusion which postulates that the oxidant flux goes from regions of high concentration to regions of low concentration, with a magnitude that is proportional to the concentration gradient [[68], [69], [70]].

In order to determine the most optimal CRBP pellets, the Kosemeyer-Peppas model was used to calculate the release rate constants of KMnO₄ for both CRBP-PEO and CRBP-PVAc pellets and the results were then compared to literature values. Table 1 describes the Korsmeyer-Peppas model parameters for both CRBP-PEO and CRBP-PVAc pellets. The 20 % and 40 % of CRBP-PVAc pellets were observed to release KMnO₄ at a faster initial rate compared to the CRBP-PEO pellets ($K_{kp}$ (CRBP-PVAc) > $K_{kp}$ (CRBP-PEO)). According to the Korsmeyer-Peppas model for polymeric structures, the oxidant transport occurs via Fickian diffusion when the value of the parameter *n* is below or equal to 0.45, an *n* value between 0.45 and 0.89 indicates anomalous (Non-Fickian) transport, while *n* values higher than 0.89 indicate super case II transport (Non-Fickian) [61,71,72]. Fickian diffusion is often used in controlled-release pellets because it results in a predictable and consistent release rate. However, non-Fickian diffusion can also be used to achieve a desired release rate, but it is less predictable and inconsistent [61]. Understanding the difference between Fickian and non-Fickian diffusion is important in the design and development of controlled-release pellets because it allows for the prediction and control of the release rate, which is crucial for achieving the desired remediation effect [61]. Based on Tables 1 and it can be observed that both CRBP-PVAc and CRBP-PEO pellets followed a non-Fickian diffusion release controlled by the relaxation of the polymer chains, which characterizes the deterioration of the CRBP pellets. Similar studies investigated model parameters ($K_{kp}$, *n*) for KMnO₄ release from comparable polymers such as paraffin wax, PMMA and polycaprolactone [33,34,73]. The present study resulted in release rate constants ($K_{kp}$ = 3.1 and $K_{kp}$ = 3.8) for 20 % and 40 % of KMnO₄-CRBP-PVAc pellets, respectively (Table 1). For a comparable oxidant-to-polymer ratio of 0.25:1 (20 wt% KMnO₄), a recent study reported $K_{kp}$ = 2.71 and *n* = 0.278 using controlled release pellets embedded in polycaprolactone (PCL) polymer while a previous study observed $K_{kp}$ = 0.052 and an n value of 0.187 for controlled release pellets using a waxy polymer matrix [33,73]. Additionally, for a lower oxidant-to-polymer ratio of 1:8, an observed a model value $K_{kp}$ = 0.0657 and n = 0.261 using KMnO₄ as the oxidant of interest and Poly (methyl methacrylate) (PMMA) as the polymer shell [34]. These minor differences in release rate constants between the encapsulated KMnO₄ particles can be attributed to the different chemical structures and physical properties of the various polymers used which influence the diffusion of oxidant outside the polymer matrix [34]. Furthermore, for the investigated CRBP-PEO and CRBP-PVAc pellets, it can be noticed that there was a different trend for $K_{kp}$ model values based on the oxidant amount and polymer type used. From Table 1, the $K_{kp}$ release rate constants were found to decrease with KMnO₄:Polymer ratio increase from 0.25:1 to 0.67:1 for CRBP-PEO pellets while it tended to increase for CRBP-PVAc pellets. It can also be observed that CRBP-PVAc pellets followed similar release rate trends as in the literature, where controlled release pellets containing KMnO₄ and encapsulated in paraffin wax matrix had an initial $K_{kp}$ = 0.052 for a Polymer: KMnO₄ ratio of 5:1 only to increase to $K_{kp}$ of 0.081 and 0.122 for ratios of 2:1 and 1:1, respectively [33].

**Table 1:** Korsmeyer-Peppas release model parameters for 20 % and 40% CRBP-PEO and CRBP-PVAc pellets.

CRBP KMnO₄ pellet types.	KMnO₄: Polymer Mass Ratio	Korsmeyer-Peppas Model Parameters		
		$K_{kp}$	n	$r^{2}$
40 wt% CRBP-PEO	0.67:1	1.38	0.755	0.98
20 wt% CRBP-PEO	0.25:1	2.09	0.661	0.97
40 wt% CRBP-PVAc	0.67:1	3.84	0.857	0.95
20 wt% CRBP-PVAc	0.25:1	3.11	0.917	0.95

By comparing the release profiles of CRBP-PEO and CRBP-PVAc pellets using calculated release models, it can be observed the CRBP-PVAc pellets yielded a higher KMnO_4_ release percentage and rate compared to CRBP-PEO pellets. The rapid initial release of the oxidant from the encapsulation matrix is an essential aspect of targeted delivery. For this reason, they were used as the main pellets for the following experiments.

##### 3.1.1.2 Kinetic release of 20 %, 40 % and 60 % of KMnO₄ in CRBP-PVAc pellets baked at 120, 140 °C for 2, 4, and 10 min in water

**Fig. 2:**
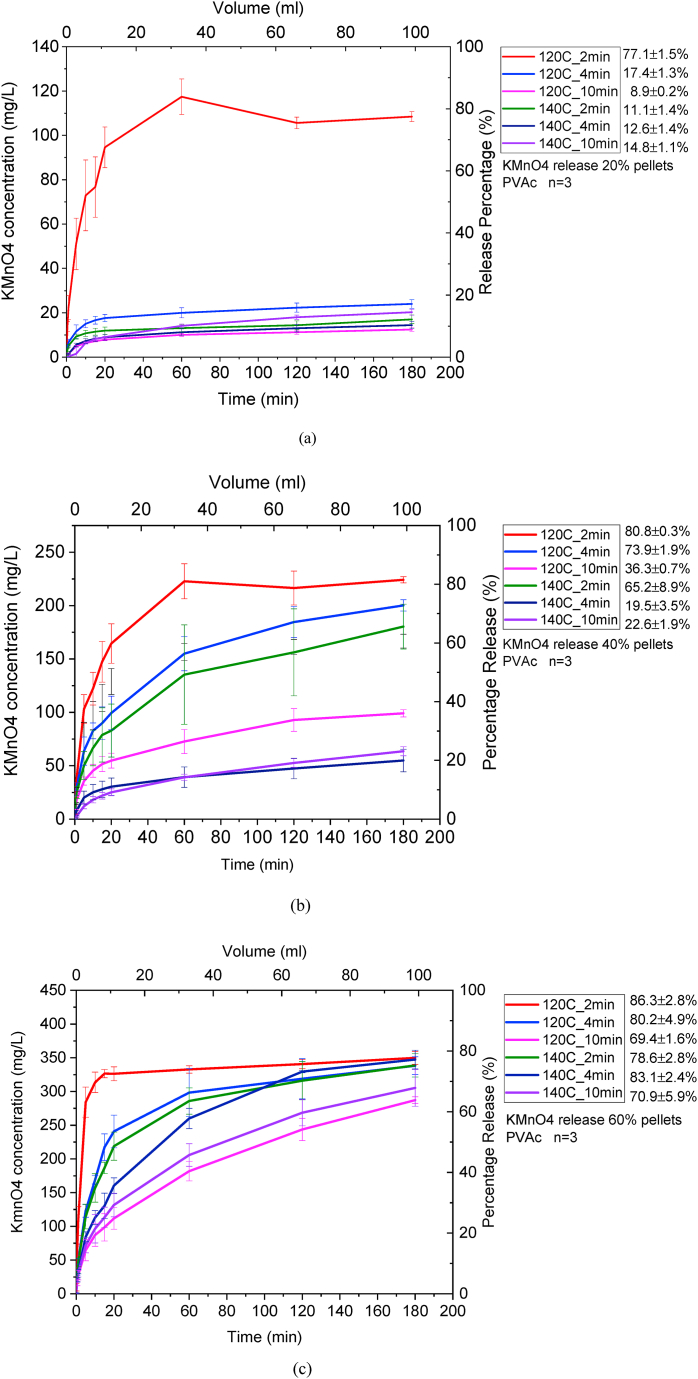
The average concentration and percentage releases in water for 20 %(a), 40%(b), and 60 %(c) KMnO_4_ CRBP-PVAc pellets baked at 120 °C and 140 °C for 2, 4, and 10 min.

The following graphs demonstrate the results obtained for CRBP-PVAc pellets:

The highest release percentages for 20 %, 40 %, and 60 % KMnO₄ CRBP-PVAc pellets were 77 %, 81 %, and 86 %, respectively, when baked for 2 min at 120 °C (Fig. 2a–c, Table S5). Overall, the 60 % of KMnO₄ CRBP-PVAc pellets baked for 2 min at 120 °C yielded the highest release percentages. It indicates that the baking temperature, time and mass ratios affect the KMnO₄ release percentage. This was also observed in other controlled release studies and can be explained by the decreasing surface porosity of the polymer matrix and changes in pellet hardness and polymer density due to the induced thermal treatment [74,75]. When CRBP pellets encapsulated in lipid (Gelucire) matrices were exposed to increasing thermal stress conditions for a prolonged period of time (24 h), they yielded lower drug release rates and percentages for all cured pellet formulations with low release percentages (85%) when exposed to high heat (45 °C) and higher release percentages (100%) when exposed to lower heat (40 °C) for all pellet formulas [76]. These observed high release percentages with decreasing temperature can be attributed to a decrease in the residual moisture content of the pellets, which leads to the decrease of the cohesive forces within the system and, thus, facilitates water and drug diffusion and accelerates pellet disintegration. Thus, curing at higher temperatures improves the mechanical stability of the polymeric matrices and improves cohesive pellet forces, leading to a lower release percentage [76]. The CRBP-PVAc pellets that were studied similarly showed that as the temperature and baking time increased (120 °C–140 °C and 2, 4, and 10 min, respectively), the pellets' internal structure changed due to the hygroscopic nature of PVAc causing water uptake. This can lead to a decrease in moisture allowing the polymer to act as plasticizer, resulting in plastic flow, deformation, and coalescence of particles [75,76].

Furthermore, the Korsmeyer-Peppas release model parameters were also calculated for the investigated CRBP-PVAc pellets and yielded results shown in Table S6. It can be observed that increasing the baking temperature from 120 °C to 140 °C and baking time from 2 to 4 min and then 10 min had a significant effect in reducing the release rate index $K_{kp}$ for the 20 %, 40%, and 60% CRBP-PVAc pellets (Table S6). For instance, the release rate constant $K_{kp}$ for 40 % KMnO₄ CRBP-PVAc pellets baked at 120 °C for 2 min decreased from 19.6 to 12.2 compared to those baked at 140 °C. Increasing the baking time from 2 to 4 and 10 min for these same 40 % KMnO₄ CRBP-PVAc pellets also reduced their release rate constants $K_{kp}$ from 19.6 to 14.2 and then 3.1, respectively. This trend is similar to what was reported for controlled-release hydrochloride (HCL) pellets containing diphenhydramine, where the release rate dissolution profile decreased with increasing curing temperature from 30 °C to 60 °C and curing time from 1 to 3 h and 5 h [77]. In addition, all CRBP-PVAc pellets studied followed a Fickian diffusion of KMnO₄ from the polymer matrix (n < 0.45). This is significant since Fickian diffusion demonstrates that the release rate mechanism of CRBP-PVAc pellets is dominated by diffusion rather than swelling [78], thus promoting a longer drug release rate due to changes in the pellet macromolecular structure induced by increasing temperatures (120°C–140 °C) contrary to the non-fiction diffusing trend observed earlier in CRBP-PVAc baked at a lower temperature (100 °C) [79]. Additionally, the CRBP-PVAc pellet release model generated $r^{2}$ values that were in the acceptable range ($r^{2}\geq 0.90$) [80]. This is similar to what previous studies observed when analyzing CRBP systems in which wax was used as an encapsulating matrix for KMnO₄ and generated $r^{2}>0.90$ values mirroring those demonstrated in Table S6 [33,81].

The release rate constant, $K_{kp}$, increases with increasing oxidant mass ratios, as observed in similar controlled release studies [33,34]. This behavior is primarily dependent on oxidant solubility, polymer type, and diffusion rate. Additionally, the weight percentage of KMnO₄ also plays a crucial role in affecting the release rate of CRPB-PVAc pellets. In this study, the 60% KMnO₄ CRBP-PVAc pellets were observed to have the highest release rate constants $K_{kp}$ across all CRBP-PVAc pellet groups, while 20% and 40% of KMnO_4_ CRBP-PVAc pellets yielded lower release rate constants (Table S6).

### 3.2 Release in soil

#### 3.2.1 Kinetic release of 60% KMnO₄ CRBP-PVAc pellets baked for 2 min at 120 °C in sand at pH = 6.0 and at pH = 8.0

**Fig. 3:**
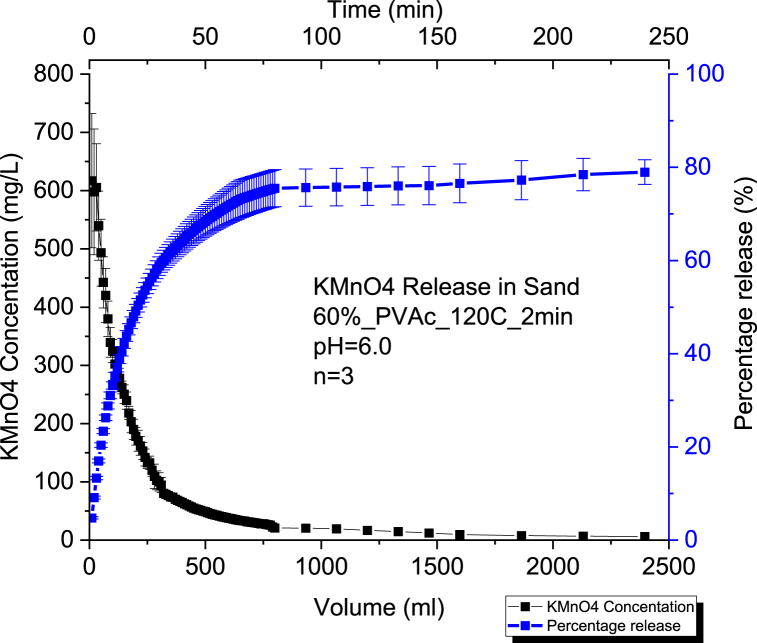
Average Concentration and Percentage Release of Triplicate Runs n = 3 using 60 % KMnO₄ - PVAc pellet baked for 2mins at a constant pH = 6.0 in a 100 % Sand by mass Soil Column Setup.

**Fig. 4:**
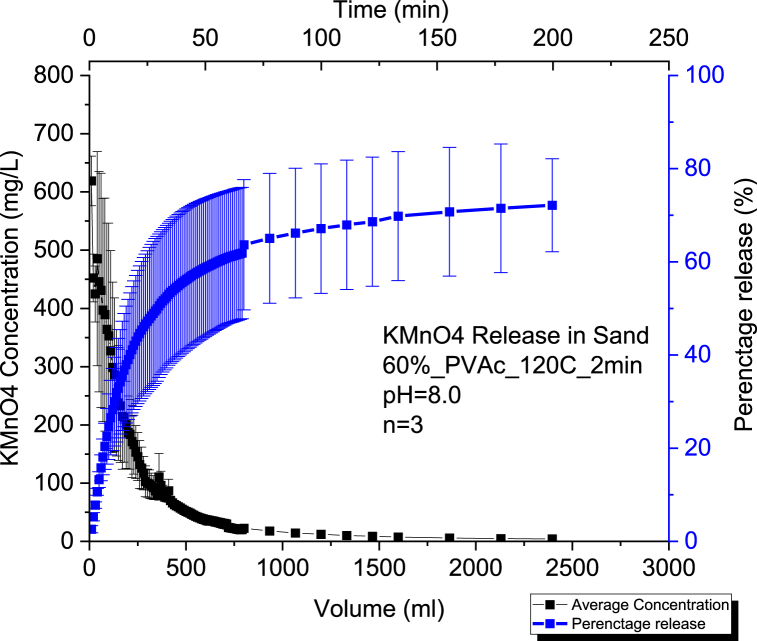
Average Concentration and Percentage Release of Triplicate Runs n = 3 using 60 % KMnO₄ - PVAc pellet baked at 120 for 2 min at a constant pH = 8.0 in a 100 % Sand by mass Soil Column Setup.

The average release percentage was 73% at pH of 6.0 and 72 % at pH of 8.0 (Fig. 3, Fig. 4). Consequently, we can notice that the change in pH within the sand media did not have a considerable effect on the KMnO₄ release for CRBP-PVAc pellets since the difference is not statistically significant (p-value >0.05) (Table S7). This is similar to what was observed for controlled release fertilizer pellets encapsulated in a K-carrageenan-based hydrogel where no significant differences were observed between pH treatments (pH = 4.5, pH = 5.5 and pH = 6.3) for the nitrogen (N) release from the encapsulated fertilizer to the aqueous medium (p > 0.05) [82]. It can be seen that the release rate of CRBP-PVAc pellets in sandy soil followed a similar biphasic release trend to what was observed in water. In Table S8, the release rate constants were $K_{kp}$ = 10.01 for pH = 6.0 and $K_{kp}$ = 10.2 for a pH = 8.0 using the Kosemeyer-Peppas model. As for the type of the release, the CRBP-PVAc pellets followed a Fickian diffusion with n < 0.45 for both pH = 6.0 and pH = 8.0. This is similar to what was observed when dealing with nitrification inhibitor Dicyandiamide (DCD) encapsulated in a biodegradable polymer poly (3-hydroxybutyrate-*co*-3-hydroxyvalerate (PHBV) where controlled release pellets tested in sandy media column showed a diffusion release process following a Fickian trend (*n* < 0.45) when fitting the obtained results to the Kosemeyer-Peppas model [83].

#### 3.2.2 Kinetic release of 60% KMnO₄ CRBP-PVAc pellets baked for 2mins at 120 °C in clay at pH = 6.0 and at pH = 8.0

**Fig. 5:**
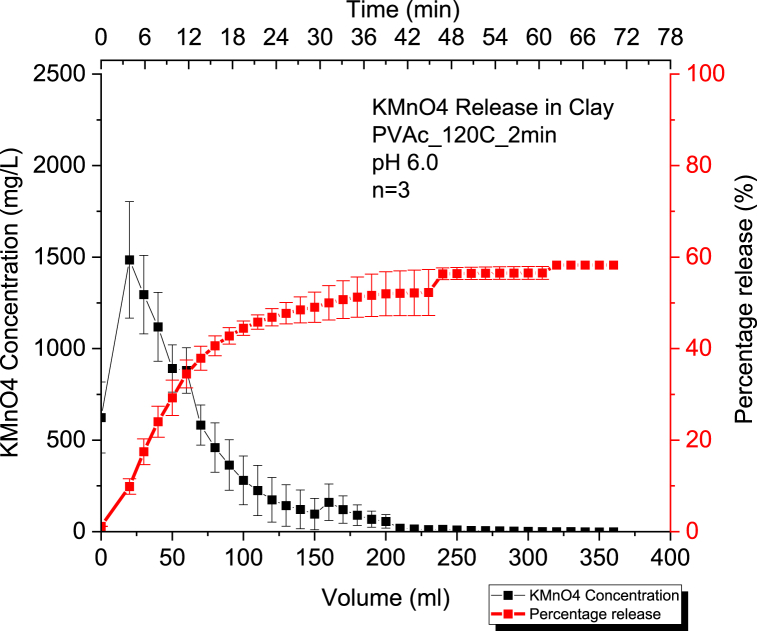
Average concentration and percentage release of KMnO₄ (n = 3) using 60 % KMnO₄ - PVAc pellet baked for 2mins at a constant pH = 6.0 in a 50 % clay and 50 % sand by mass soil column setup.

**Fig. 6:**
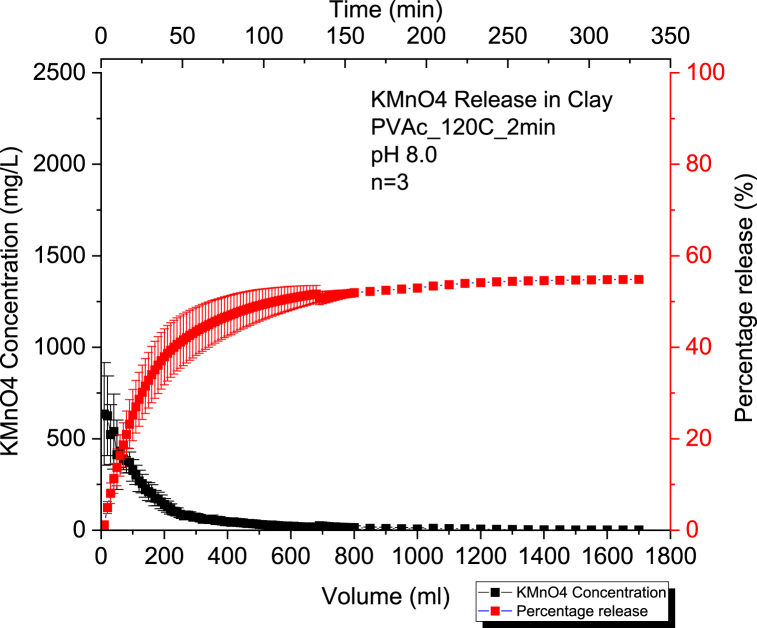
Average concentration and percentage release of KMnO₄ (n = 3) using 60 % KMnO₄ - PVAc pellet baked for 2 min at a constant pH = 8.0 in a 50 % clay and 50 % sand by mass soil column setup.

We can observe that the percentage release of potassium permanganate is 58 % at a pH of 6.0 and 55 % at a pH of 8.0 for 60 % KMnO₄ CRBP-PVAc pellets baked at 120 °C for 2 min (Fig. 5, Fig. 6). The change in pH from 8.0 to 6.0 did not affect CRBP-PVAc pellet release significantly (p > 0.05) (Table S9). Similar to release in sand, the Kosemeyer-Peppas model results shown in Table S8 demonstrated a Fickian diffusion release for the CRBP-PVAc pellets with *n* < 0.45 at both pH = 6.0 and pH = 8.0. Likewise, the release rate of fertilizer in SCMC-g-poly (AA), polyvinylpyrrolidone (PVP) and silica particles was similar to that of CRBP-PVAc in soil, with *n* values of 0.297, 0.345, and 0.342 respectively [84]. This is a reflective of a Fickian diffusion type (n < 0.45), and is similar to what was observed in the present study for the investigated CRBP-PVAc pellets [84]. However, the observed release rate constants of CRBP-PVAc pellets in clay were lower than in sand at pH 6.0 ($K_{kp\;\left(Clay\right)}$ = 10.01 > $K_{kp\left(Sand\right)}$ = 9.8) and at pH = 8.0 ($K_{kp\;\left(clay\right)}=$ 10.2 > $K_{kp\left(Sand\right)}$ = 9.9) (Table S8). This is mainly due to the clay's higher porosity and lower permeability [85]. Results also showed no significant difference in the release rate constants at pH = 6.0 and pH = 8.0 (Table S8).

#### 3.2.3 Kinetic release of 60 % KMnO₄ CRBP-PVAc pellets baked for 2 min at 120 °C in silt at pH = 6.0 and at pH = 8.0

**Fig. 7:**
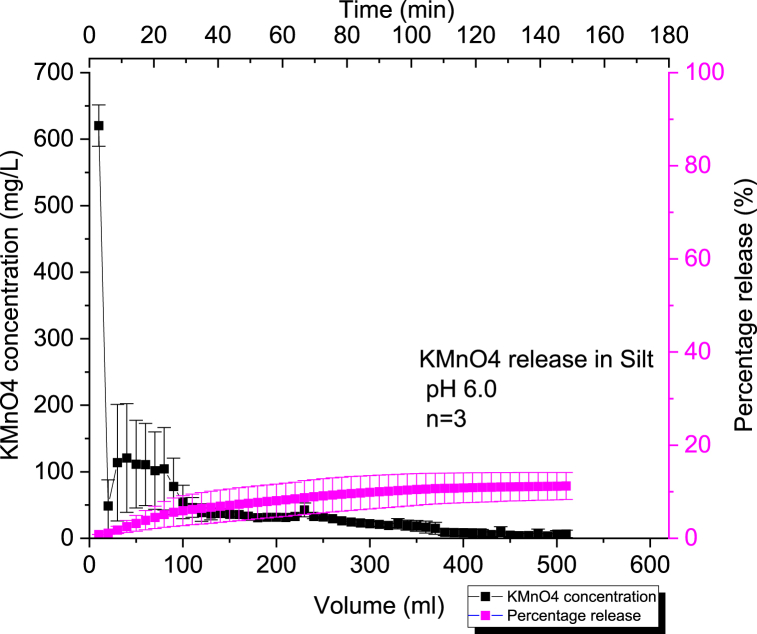
Average concentration and percentage release of KMnO4 (n = 3) using 60 % KMnO₄- PVAc pellet baked for 2mins at a constant pH = 6.0 in a 50 % silt and 50 % sand by mass soil column setup.

**Fig. 8:**
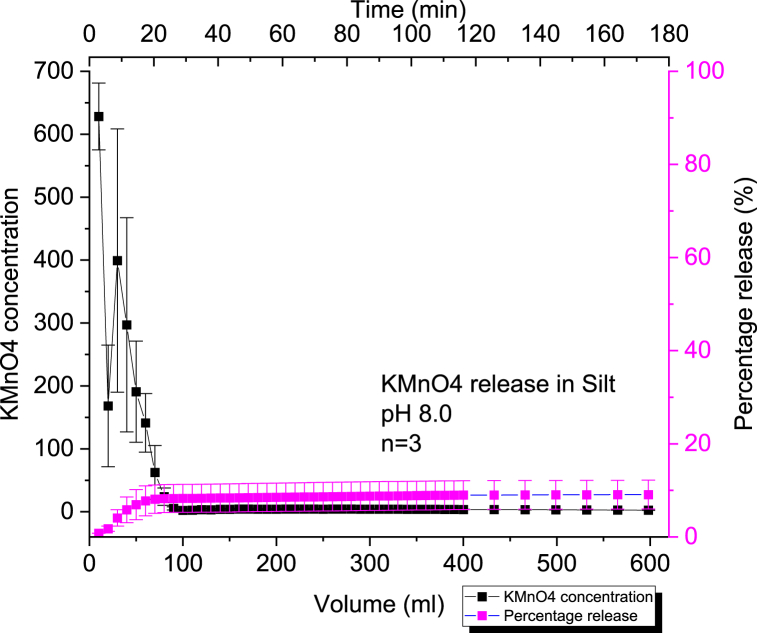
Average concentration and percentage release of KMnO4 (*n* = 3) using 60 % KMnO₄ - PVAc pellet baked for 2 min at a constant pH = 8.0 in a 50 % silt and 50 % sand by mass soil column setup.

We can observe that the percentage release of KMnO_4_ within the silt columns was very low (10 % for pH = 6.0 and 9 % for pH = 8.0) compared to the release in clay or sand (Fig. 7, Fig. 8). XPS analysis on silt soil samples demonstrated the formation of MnO_2_ at the bottom of the column (Figs. S9 and S10). This low-release behavior observed in the silt column experiments can be related to the acute and significant consumption of KMnO_4_ by NOM present in the silt column through oxidation as there was noticeable dark brown MnO_2_ formation (Fig. S8). A theoretical stoichiometric relationship was previously demonstrated, and describes the oxidation of permanganate with NOM yielding MnO_2_, CO_2_ and H_2_O [86]:

(13) $$28MnO_{4}^{-}+3C_{7}H_{8}O_{4}+28H^{+}\to 28MnO_{2}+21CO_{2}+26H_{2}O$$

The transport of potassium permanganate in low permeability silty soil media was found to result in similar consumption of KMnO_4_ in the presence of NOM and formation of MnO_2_ upon visual and XPS examination (Fig. S8, S9 and S10) [87]. This is due to the co-diffusion of the $K^{+}$ cations with present solute samples while the $\text{Mn}O_{4}^{-}$ was reduced to MnO_2_ during oxidation. Other studies, have also reported similar consumption phenomena when describing the presence of KMnO_4_ within soil samples containing high NOM [[88], [89], [90], [91]]. It can also be seen that the change in pH within the silt media did not have a considerable effect on the KMnO₄ release for CRBP-PVAc pellets since the difference is not statistically significant (p-value >0.05) (Table S10).

Similar to controlled release in sand and clay, the CRBP-PVAc pellets demonstrated to follow the Kosemeyer-Peppas model with good statistical variance ($r^{2}\geq 0.90$) as seen in Table S8. The CRBP-PVAc still followed a Fickian diffusion release type with *n* < 0.45 at both pH = 6.0 and pH = 8.0 (Table S8). This was observed in both diffusive transport and bench scale investigations of permanganate natural oxidant demand kinetics (NOD) as NOD increases within soil columns [87,88].

#### 3.2.4 Longevity of 60% KMnO_4_ CRBP-PVAc pellets baked for 2 min at 120 °C in soil

From the experimental results, it is evident that the longevity of the release material (KMnO_4_) varies with the soil type and the pH of the environment. In sand, where the porosity is 0.5, about 72–73 % of the KMnO_4_ is released over 65.79 pore volumes of water at both pH 6.0 and 8.0 (Table S11). This indicates a relatively high longevity of the release material in sandy soils which can be attributed to the larger pore volume facilitating the movement of water and hence the release of KMnO_4_ from the pellets [[92], [93], [94], [95]].

In contrast, the release efficiency is lower in clay (55–58 % over 7.83–29.35 pore volumes) and even lower in silt (9–10 % over 12.59–14.81 pore volumes) (Table S11). This suggests that the longevity of the KMnO_4_ release is reduced in clay and silt soils compared to sand, due to the smaller pore volumes observed in these soil types and higher organic matter content observed in silty soils, which consumes KMnO_4_ [88,[96], [97], [98]]. Additionally, the lower permeability of both silty and clay soils restricts the movement of water and hence the release of KMnO4 from the pellets [98,99]. This is similar to what was observed when optimizing slow-release permanganate gel (SRPG) for KMnO_4_ release in soil column tests with low permeability media resulting in diminished release potential of SRPG solutions [98]. Importantly, the results also indicate that the pH of the environment does not substantially affect the longevity of the studied 60% CRBP-PVAc pellets, as the release efficiencies were comparable at both pH 6.0 and 8.0 for all soil types.

In summary, the longevity of the KMnO_4_ release from the 60 % CRBP-PVAc pellets is highest in sand and decreases in the order sand > clay > silt. This is an important consideration for the application of this technology in different soil types and indicates that the CRBP pellets may be most effective in sandy soils or soils with low organic matter content.

### 3.3 Release in a mix of soil and water

#### 3.3.1 Kinetic release of 60 % KMnO₄ CRBP-PVAc pellets baked for 2 min at 120 °C in a mix of soil and water

**Fig. 9:**
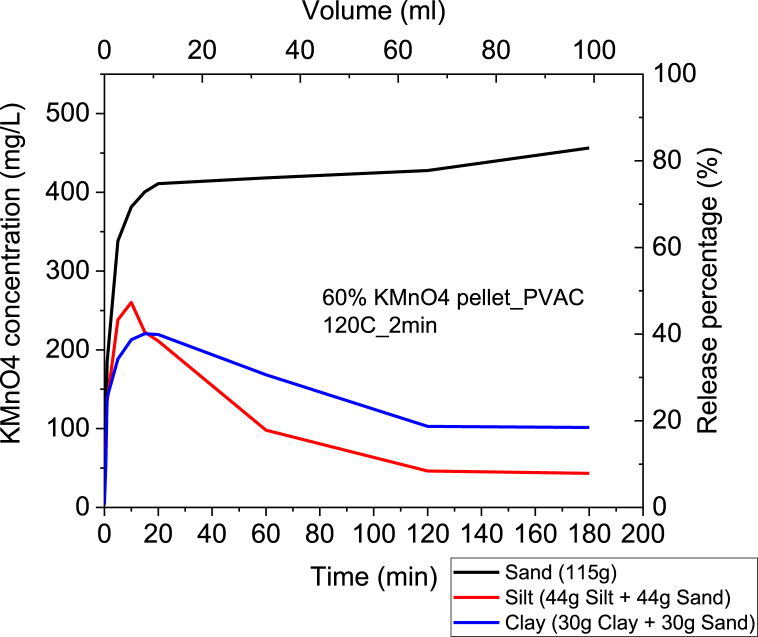
Batch Tests with Kinetic Release of KMnO₄ in a mix of Soil and Water for 60 %- KMnO₄ PVAc Baked pellets at 120 °C.

The release percentage of KMnO_4_ in the mix of silt and water was observed to be lower than those in a mix of clay and sand with water, with a 9% of release percentage compared to 18 % and 81 %, respectively (Fig. 9). Similar to the release in silt columns, these results confirm that KMnO_4_ was consumed by NOM present in the mix of silt and water, indicating the oxidation of KMnO_4_. This finding is supported by literature suggesting that the presence of natural organic matter in soil can reduce the effectiveness of controlled-release pellets especially for KMnO_4_ used as an oxidant [96], as it can consume the active ingredient before it can be released. However, other literature has shown that using PVAc as an embedding polymer can increase the effectiveness of controlled-release pellets in different soil types, including sandy soils [89]. Thus, controlled-release pellets using PVAc as an embedding polymer are most effective within the mix of sand and water and should be implemented in groundwater tables that meet these soil specifications.

## 4 Conclusion

This study investigated the feasibility of KMnO_4_ in biodegradable polymers (PVAc and PEO) by producing CRBP pellets with 20 %, 40 %, and 60 % of KMnO_4_ to polymer mass ratios. It was found that increasing the heating temperature and baking time reduced the percentage release of KMnO_4_ from the CRBP pellets. Furthermore, CRBP pellets embedded in PVAc were found to be more effective than those embedded in PEO due to their higher release percentage and rate. In water batch tests, the 60 % of KMnO_4_ CRBP-PVAc pellets baked at 120 °C for 2 min demonstrated the highest release percentage of 86 %. In soil column release experiments, the pH changes did not significantly affect the release behavior and percentages of CRBP-PVAc pellets cooked at 120 °C for 2 min in sand, clay, and silt media. However, KMnO_4_ release was highest in sand media, with 73 % at a pH of 6.0 and 72 % at a pH of 8.0, while silt media had a significantly lower release of KMnO_4_ (10 % at a pH of 6.0 and 9 % at a pH of 8.0) likely due to the consumption of KMnO_4_ by NOM present in the collected silt. As for clay, the release percentage was 58% at pH = 6.0 and 55 % at pH = 8.0. Additionally, it was found that all CRBP-PVAc pellets followed a biphasic release behavior with Fickian/non-Fickian diffusion type across both water and soil release experiments, which is consistent with other literature studies (Table S12). A mix of water and soil batch tests also demonstrated a significant consumption of KMnO_4_ had occurred in a mix of silt and water with CRBP-PVAc pellets releasing only 15 % of KMnO_4_, while those CRBP-PVAc pellets within a mix of clay and water demonstrated a 32 % KMnO_4_ release and pellets in a mix of sand and water showed the highest release with a percentage of 71 %.

The Korsmeyer-Peppas model was effective in describing the release rate behavior of both CRBP-PVAc and CRBP-PEO pellets. It was determined that the release rate constant $K_{kp}$ was dependent on the different pellet baking temperatures and time, and that most CRBP pellets observed a Fickian diffusion release type especially those embedded in PVAc. The data from batch tests in both water and soil columns suggests that the CRBP-PVAc pellets can deliver KMnO_4_ at a controlled rate over a prolonged period in both batch and continuous flow systems.

The consideration of site treatment and selection should include factors such as NOM, NOD, pH, water chemistry, presence of pollutants, and soil screening/characterization. Other crucial parameters for CRBP pellet development include temperature, cooking time, oxidant-to-polymer mass ratio, and potential interaction with other pollutants. The long-term stability and cost-effectiveness of CRBP treatment should also be evaluated, as well as the potential for secondary pollutants and use in conjunction with other remediation technologies. Regular monitoring and evaluation through soil and water analysis and a multidisciplinary approach are recommended.

## Data availability statement

Data included in article/supp. Material/referenced in article.

## CRediT authorship contribution statement

**Mehdi Lamssali:** Formal analysis, Investigation, Methodology, Writing – original draft. **Stephanie Luster-Teasley:** Conceptualization, Formal analysis, Funding acquisition. **Dongyang Deng:** Conceptualization, Formal analysis, Methodology, Writing – original draft, Writing – review & editing. **Nafisa Sirelkhatim:** Methodology, Writing – review & editing. **Yen Doan:** Methodology. **Mosarrat Samiha Kabir:** Investigation, Methodology. **Qingan Zeng:** Methodology, Writing – review & editing.

## Declaration of competing interest

The authors declare that they have no known competing financial interests or personal relationships that could have appeared to influence the work reported in this paper.
