# Supplementary material for: Release efficiencies of potassium permanganate controlled-release biodegradable polymer (CRBP) pellets embedded in polyvinyl acetate (CRBP-PVAc) and polyethylene oxide (CRBP- PEO) for groundwater treatment
Source: Heliyon. 2023 Oct 10;9(10):e20858. doi: 10.1016/j.heliyon.2023.e20858 (PMC10585301; doi:10.1016/j.heliyon.2023.e20858)
Supplement: Multimedia component 1 [file mmc1.docx]

# Supplementary Material

**Release Efficiencies of Potassium Permanganate Controlled-Release Biodegradable Polymer (CRBP) Pellets Embedded in Polyvinyl Acetate (CRBP-PVAc) and Polyethylene Oxide (CRBP- PEO) for Groundwater Treatment**

Mehdi Lamssali^a^, Stephanie Luster-Teasley^b,*^, Dongyang Deng^c,*^, Nafisa Sirelkhatim^d^, Yen Doan^e^, Mosarrat Samiha Kabir^f^, Qingan Zeng^g^

^a^ Department of Applied Science and Technology, North Carolina A&T State University, 27411 Greensboro, North Carolina, United States. Email: [mlamssali@aggies.ncat.edu](mailto:mlamssali@aggies.ncat.edu)

^b^ Provost and VC for Academic Affairs, North Carolina A&T State University, 27411 Greensboro, North Carolina, United States. Email: luster@ncat.edu

^c^ Department of Built Environment, North Carolina A&T State University, 27411 Greensboro, North Carolina, United States. Email: [ddeng@ncat.edu](mailto:ddeng@ncat.edu)

^d^ Dean’s Office, Joint School of Nanoscience and Nanoengineering, 27401 Greensboro, North Carolina, United States. Email: [nhsirelk@ncat.edu](mailto:nhsirelk@ncat.edu)

^e^ Department of Civil and Environmental Engineering, North Carolina A&T State University, 27411 Greensboro, North Carolina, United States. Email: [ytdoan@aggies.ncat.edu](mailto:ytdoan@aggies.ncat.edu)

^f^ Department of Nanoengineering, Joint School of Nanoscience and Nanoengineering, 27401 Greensboro, North Carolina, United States. Email: [mkabir1@aggies.ncat.edu](mailto:mkabir1@aggies.ncat.edu)

^g^ Department of Computer Systems Technology, North Carolina A&T State University, 27411 Greensboro, North Carolina, United States. Email: [qzeng@ncat.edu](mailto:qzeng@ncat.edu)

**Corresponding Authors.** Email addresses: luster@ncat.edu (S. Luster-Teasley), [deng@ncat.edu](mailto:deng@ncat.edu) (D. Dongyang).

## **Figures**

**Fig. S1.** Clay collection area and location profile
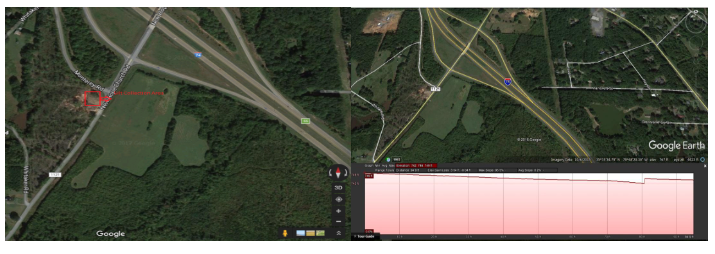

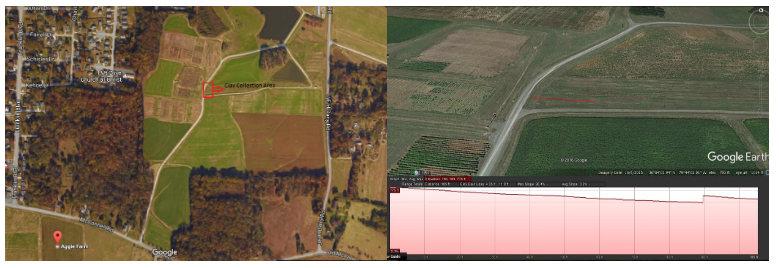


**Fig. S2.** Silt collection area and location profile

**
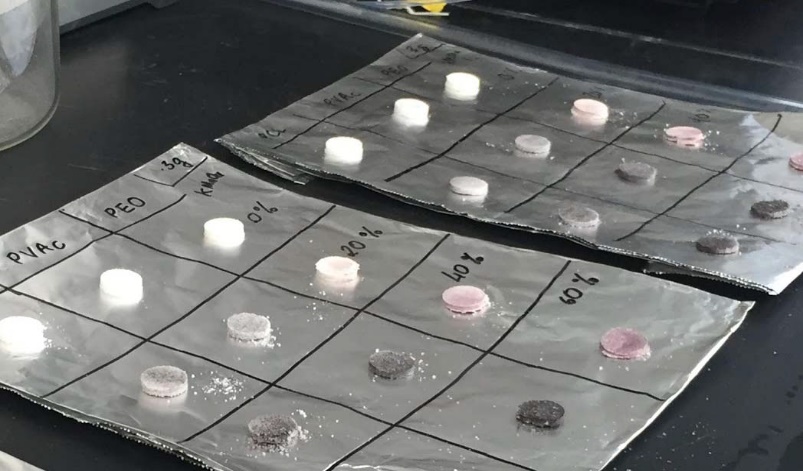
(a) (b)**

**Fig. S3.** Controlled-Release Biodegradable Polymer (CRBP) Pellets before (a) and after (b) experimental runs.

**Fig. S4.** Schematic Diagram demonstrating the Preparation Process of Controlled-Release Biodegradable Polymer (CRBP) Pellets.


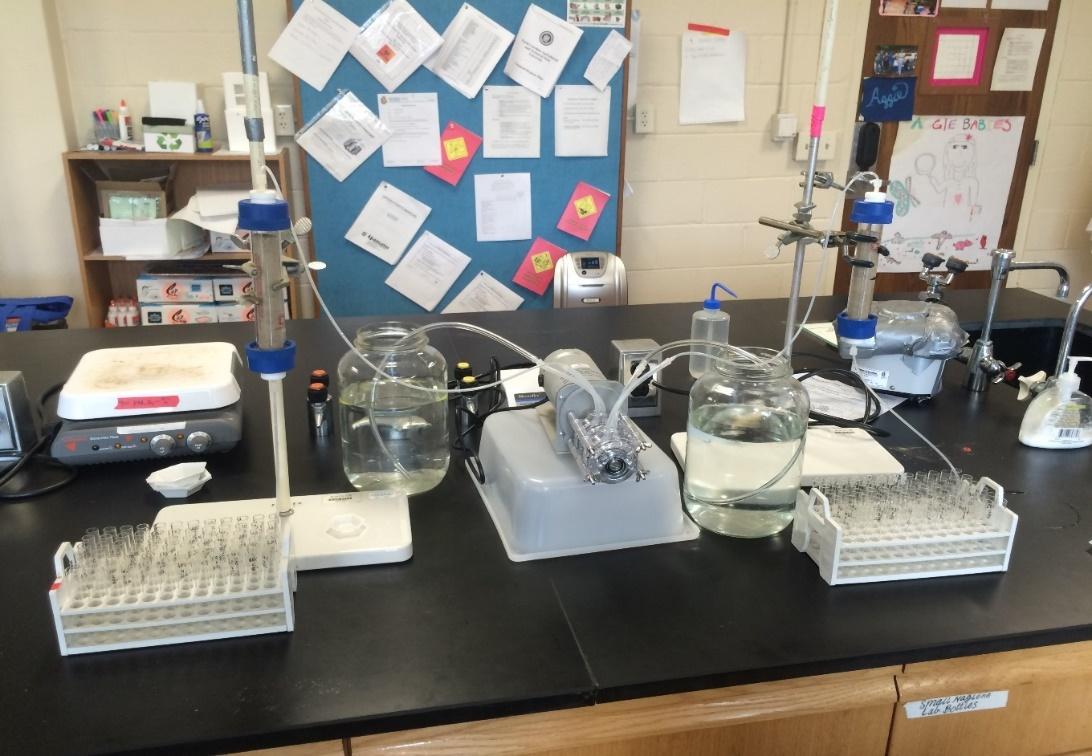


**Fig. S5.** Soil Column experimental setup


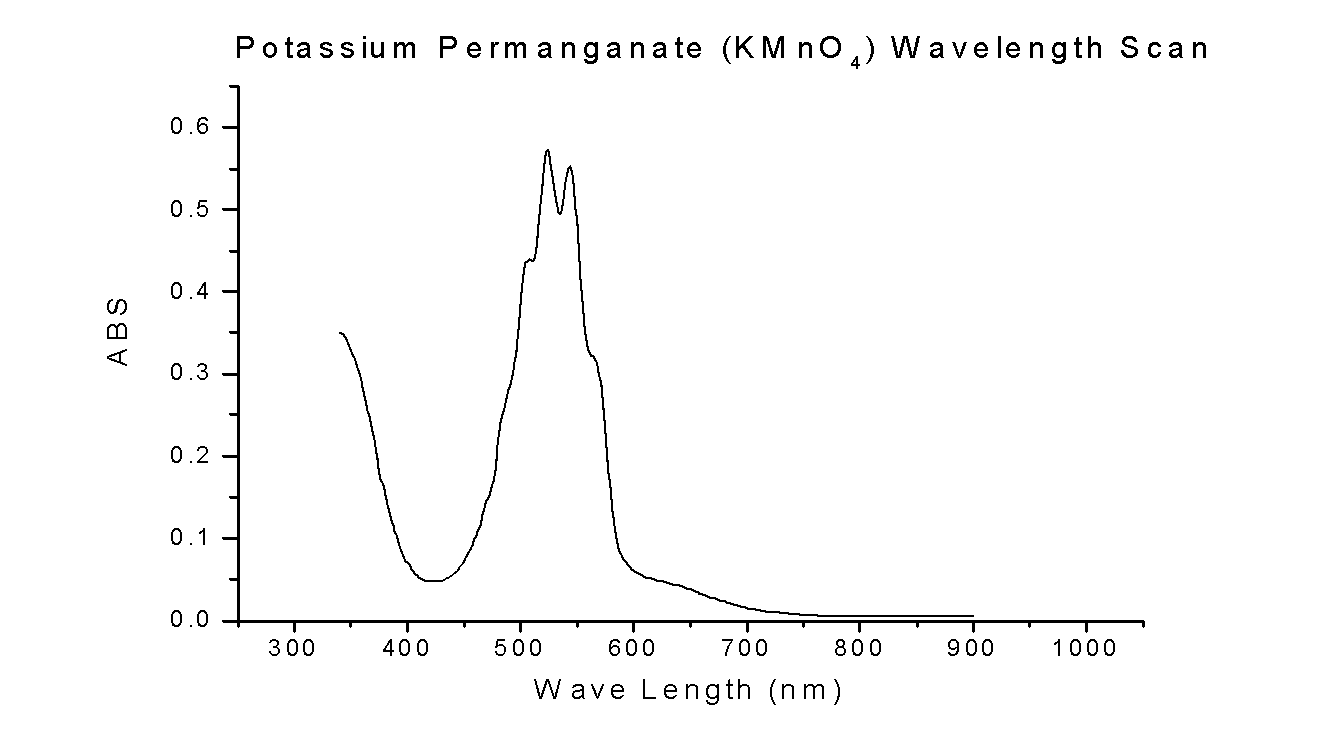


**Fig. S6.** Potassium Permanganate (KMnO_4_) wavelength scan showing the maximum absorbance at λ=525nm.


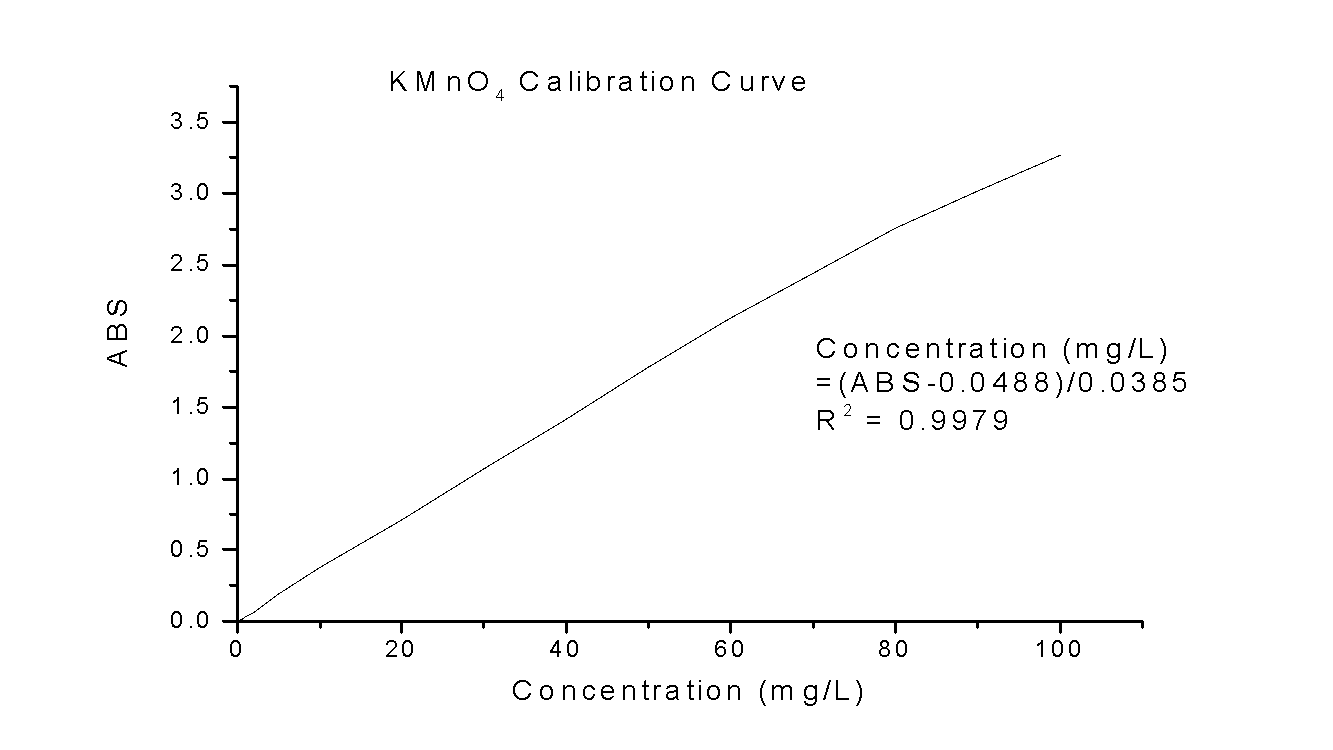


**Fig S7.** KMnO₄ calibration curve for absorbance at 525 nm


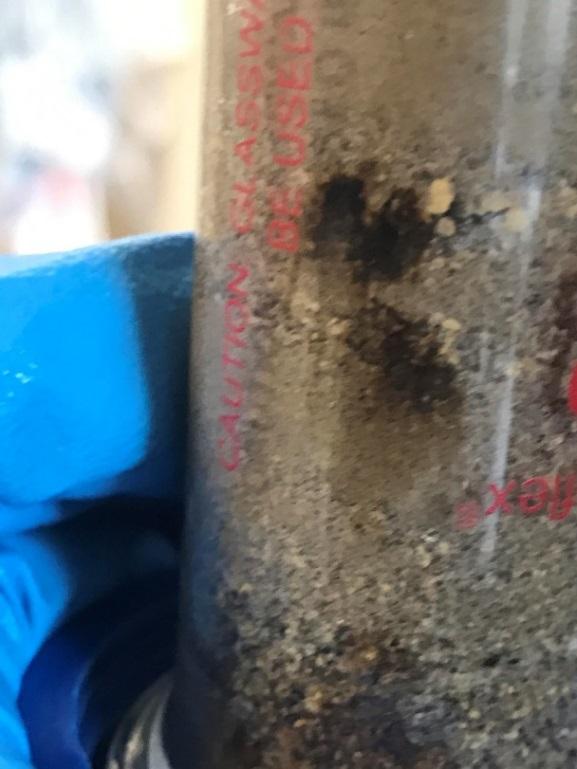

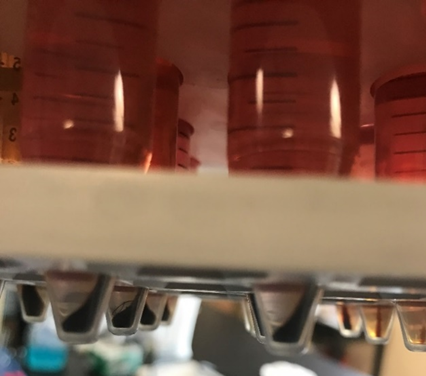


1. **(b)**

**Fig. S8.** Soil column with MnO₂ formation (Left) and sample tubes collected from silt soil columns with MnO₂ formation at the bottom (Right)


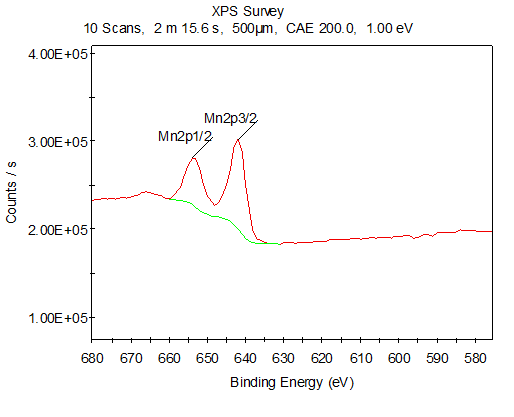


**Fig. S9.** XPS Photoelectron survey spectrum of silt Soil after treatment by 60 % KMnO_4_ CRBP-PVAc pellets demonstrating the presence of manganese between binding energy of 630 and 660 eV


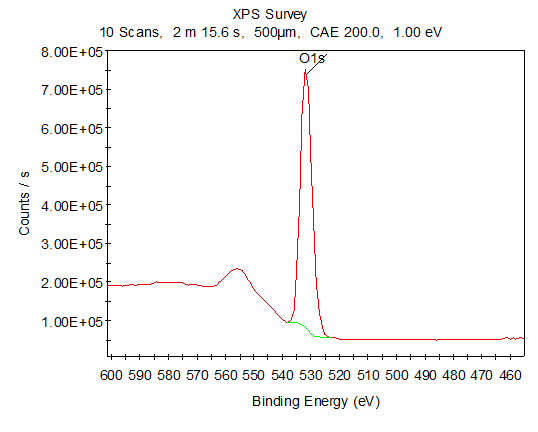


**Fig. S10.** XPS Photoelectron survey spectrum of silt soil after treatment by 60 % KMnO_4_ CRBP-PVAc pellets demonstrating the presence of manganese between binding energy of 630 and 660 eV

XPS spectra is shown in both figures S7 and S8, it demonstrates the presence of multiple-split peak components with two maximum peaks between binding energies of 635 and 660 eV representing Mn2p1/2 and Mn2p3/2 peak positions. Figure S9 also shows the presence of oxygen in the collected soil samples with a maximum sample peak between binding energies 525 and 540 ev representing O1s. This is similar to results obtained when analyzing MnO_2_ samples using XPS and demonstrating Mn 2p peaks and O1s at comparable range of binding energies [1].

## **Tables**

**Table S1.** CRBP Pellet Characteristics

| CRBP Pellets Polymer | KMnO₄ to Polymer mass ratio (wt%) | Baking Temperature ( °C) | Time (mins) |
| --- | --- | --- | --- |
| PEO | 20 and 40 % | 100 °C | 10 mins |
| PVAc | 20 and 40 % | 100 °C | 10 mins |
| PVAc | 20, 40 and 60 % | 120 and 140 °C | 2, 4 and 10 mins |

**Table S2.** Mass of KMnO₄ and PEO/ PVAc in CRBP based on wt% Percentages

| CRBP Pellets | 0% KMnO₄ concentration | 20 % KMnO₄ concentration | 40 % KMnO₄ concentration | 60 % KMnO₄ concentration |
| --- | --- | --- | --- | --- |
| Mass of KMnO₄ powder (grams) | 0 g | 0.06 g | 0.12 g | 0.18 g |
| Mass of PEO or PVAc powder (grams) | 0.3 g | 0.24 g | 0.18 g | 0.12 g |

**Table S3.** Kinetic release of CRBP Pellets embedded in PEO and containing 20% and 40 % KMnO₄.

| KMnO_4_ - PEO CRBP pellets | Baking time of the CRBP pellets (minutes) | Baking temperature of the CRBP pellets, 100 ºC |
| --- | --- | --- |
|  |  | Kinetic Release (n=3) |
| 20 % | 10 mins | 59.68 ± 6.61 % |
| 40 % | 10 mins | 60.84 ± 0.95 % |

**Table S4.** Kinetic release of CRBP Pellets embedded in PVAc, containing 20% and 40 % KMnO₄ and cooked for 10 mins at 100 ºC

| KMnO_4_ - PVAc CRBP pellets | Baking time of the CRBP pellets (minutes) | Baking temperature of the CRBP pellets, 100 ºC |
| --- | --- | --- |
|  |  | Kinetic Release (n=3) |
| 20 % | 10 mins | 79.97 ± 2.73 % |
| 40 % | 10 mins | 75.61 ± 2.51 % |

**Table S5.** Kinetic Release for CRBP-PVAC pellets containing 20 %, 40 %, and 60 % KmnO4 at baking temperatures of 120 and 140 ºC for 2, 4 and 10 mins respectively.

| KMnO_4_ - PVAc CRBP pellets | Baking time of the CRBP pellets (minutes) | Baking temperature of the CRBP pellets, 120ºC | Baking temperature of the CRBP pellets, 140ºC |
| --- | --- | --- | --- |
|  |  | Kinetic Release  n=3 | Kinetic Release  n=3 |
| 20% | 2 | 77.12±1.54 | 12.65±1.37 |
|  | 4 | 17.44±1.26 | 11.12±1.43 |
|  | 10 | 8.90±0.23 | 14.83±1.13 |
| 40% | 2 | 80.83±0.3 | 65.20±8.98 |
|  | 4 | 73.9±1.91 | 19.5±3.54 |
|  | 10 | 36.33±0.73 | 22.61±1.87 |
| 60% | 2 | 86.31±2.8 | 78.61±2.84 |
|  | 4 | 80.17±4.93 | 83.09±2.44 |
|  | 10 | 69.04±1.58 | 70.93±5.96 |

**Table S6.** Korsmeyer-Peppas release model parameters for 20%, 40 %, and 60% CRBP-PVAc pellets baked at 120ºC and 140ºC for 2, 4, and 10 mins

| KMnO_4_ - PVAc CRBP pellets | Baking time of the CRBP-PVAc pellets (minutes) | Baking temperature of the CRBP-PVAc pellets, 120ºC | | | Baking temperature of the CRBP-PVAc pellets, 140ºC | | |
| --- | --- | --- | --- | --- | --- | --- | --- |
|  |  | $K_{kp}$ | $n$ | $r^{2}$ | $K_{kp}$ | $n$ | $r^{2}$ |
| 20% | 2 | 17.88 | 0.447 | 0.98 | 5.31 | 0.168 | 0.93 |
|  | 4 | 7.08 | 0.185 | 0.94 | 2.85 | 0.274 | 0.91 |
|  | 10 | 2.39 | 0.266 | 0.92 | 1.43 | 0.412 | 0.94 |
| 40% | 2 | 19.61 | 0.437 | 0.98 | 12.18 | 0.355 | 0.99 |
|  | 4 | 14.17 | 0.353 | 0.99 | 4.83 | 0.272 | 0.97 |
|  | 10 | 3.06 | 0.275 | 0.98 | 2.53 | 0.424 | 0.99 |
| 60% | 2 | 31.14 | 0.388 | 0.97 | 16.84 | 0.361 | 0.97 |
|  | 4 | 14.46 | 0.427 | 0.99 | 7.70 | 0.441 | 0.98 |
|  | 10 | 8.72 | 0.404 | 0.99 | 7.37 | 0.413 | 0.99 |

**Table S7.** One-Way ANOVA analysis for 60 % CRBP-PVAc KMnO₄ concentrations within the Sand Soil media for pH=8.0 and pH=6.0.

| Source of Variation | SS | df | MS | F | P-Value |
| --- | --- | --- | --- | --- | --- |
| Between Groups | 39479.11 | 1 | 39479.11 | 1.437888 | 0.232451 |
| Within Groups | 3953710 | 144 | 27456.32 |  |  |
| Total | 3993189 | 145 |  |  |  |

**Table S8.** Korsmeyer-Peppas release model parameters for 60% CRBP-PVAc pellets baked at 120ºC for 2 mins in sand, clay and silt media at pH=8.0 and pH= 6.0

| KMnO_4_ - PVAc CRBP pellets | pH in Soil Column | Sand Media | | | Clay Media | | | Silt Media | | |
| --- | --- | --- | --- | --- | --- | --- | --- | --- | --- | --- |
|  |  | $K_{kp}$ | $n$ | $r^{2}$ | $K_{kp}$ | $n$ | $r^{2}$ | $K_{kp}$ | $n$ | $r^{2}$ |
| 60% CRBP-PVAc baked at 120ºC for 2 mins | 8.0 | 10.222 | 0.371 | 0.96 | 9.88 | 0.340 | 0.91 | 3.741 | 0.190 | 0.94 |
|  | 6.0 | 10.013 | 0.380 | 0.98 | 9.802 | 0.435 | 0.91 | 1.133 | 0.442 | 0.96 |

**Table S9.** One-Way ANOVA analysis for 60 % CRBP-PVAc KMnO₄ concentrations within the Clay Soil media for pH=8.0 and pH=6.0.

| Source of Variation | SS | df | MS | F | P-Value |
| --- | --- | --- | --- | --- | --- |
| Between Groups | 67336.31 | 1 | 67336.31 | 0.580852 | 0.448539 |
| Within Groups | 8114874 | 70 | 115926.8 |  |  |
| Total | 8182211 | 71 |  |  |  |

**Table S10.** One-Way ANOVA analysis for 60 % CRBP-PVAc KMnO₄ concentrations within the Silt Soil media for pH=8.0 and pH=6.0.

| Source of Variation | SS | df | MS | F | P-Value |
| --- | --- | --- | --- | --- | --- |
| Between Groups | 4282.003 | 1 | 39479.11 | 1.585391 | 0.210127 |
| Within Groups | 370025.1 | 137 | 2700.913 |  |  |
| Total | 374307.1 | 138 |  |  |  |

**Table S11.** KMnO_4_ Release Efficiency and Pore Volumes in Different Soil Types.

| CRBP Pellet | Soil Type | pH | Total Volume of Water Passed (ml) | Release Efficiency (%) | Number of Pore Volumes |
| --- | --- | --- | --- | --- | --- |
| 60 % CRBP-PVAc Pellets | Sand | 6.0 | 2400 | 73 | 65.79 |
|  | Sand | 8.0 | 2400 | 72 | 65.79 |
|  | Clay | 6.0 | 360 | 58 | 7.83 |
|  | Clay | 8.0 | 1700 | 55 | 29.35 |
|  | Silt | 6.0 | 510 | 10 | 12.59 |
|  | Silt | 8.0 | 600 | 9 | 14.81 |

**Table S12.** Comparative Overview of KMnO₄ Release Efficiency Among Different Encapsulation Materials Across Studies

| Reference | Encapsulated Material | Encapsulating/Binding Material Used | Composition | Key Findings |
| --- | --- | --- | --- | --- |
| This paper | KMnO_4_ | PVAc (Polyvinyl Acetate) and PEO (Polyethylene Oxide) | 20 wt%, 40 wt%, 60 wt% KMnO₄. | Biphasic and high release efficiency at 60% KMnO4. |
| [2] | KMnO_4_ | PCL (Polycaprolactone) | 1:5 KMnO_4_ to PCL | Biphasic release behavior with controlled diffusion. |
| [3] | KMnO_4_ | PMMA (Poly (methyl methacrylate)) | 2:1, 4:1, 8:1 PMMA to KmnO4 | Biphasic and controlled dissolution of oxidant |
| [4] | KMnO_4_ | Paraffin wax, quartz sand and activated carbon | 2:10:10:2 KMnO_4_: paraffin, quartz sand and activated carbon. | Biphasic release and varied remediation efficiency. |
| [5] | KMnO_4_ | Paraffin Wax | 1:1, 2:1 and 5:1 Parrafin Wax to KMnO_4_ | Biphasic release behavior with controlled diffusion. |
| [6] | KMnO_4_ | Blend of Boler way, Piccolyte resin S115, Epolene C-16, and Clorez 700 | 0.25 – 0.50 KMnO_4_ to Polymer | Biphasic release behavior with TCE reduction. |
| [7] | KMnO_4_ | PCL (Polycaprolactone) and Starch | 1.14:2:0.96 PCL, KMnO_4_ and starch. | Biphasic release behavior with TCE reduction. |

## **References**

[1] M.A. Stranick, MnO2 by XPS, Surface Science Spectra 6(1) (1999) 31-38. <https://doi.org/10.1116/1.1247888>.

[2] N.S. King, S. Luster-Teasley, C.J. Clark, Preliminary Analyses of Controlled Release of Potassium Permanganate Encapsulated in Polycaprolactone, Journal of Water Resource and Protection 13(01) (2021) 32-43. <https://doi.org/10.4236/jwarp.2021.131003>.

[3] J.O. Ighere, R.C. Chawla, Controlled-Release Analysis of Potassium Permanganate Using PMMA Matrix, Journal of Minerals and Materials Characterization and Engineering 02(06) (2014) 539-544. <https://doi.org/10.4236/jmmce.2014.26055>.

[4] H. Chen, J. Lu, L. Wu, T. Wang, Z. Liu, C. Wang, K. Yan, Developing a new controlled-release KMnO4 for groundwater DNAPL remediation, Environmental Technology & Innovation 24 (2021) 102064. <https://doi.org/10.1016/j.eti.2021.102064>.

[5] N. Kang, I. Hua, P.S.C. Rao, Production and Characterization of Encapsulated Potassium Permanganate for Sustained Release as an in Situ Oxidant, Industrial & Engineering Chemistry Research 43(17) (2004) 5187-5193. <https://doi.org/10.1021/ie0499097>.

[6] C. Ross, L.C. Murdoch, D.L. Freedman, R.L. Siegrist, Characteristics of Potassium Permanganate Encapsulated in Polymer, Journal of Environmental Engineering 131(8) (2005) 1203-1211. <https://doi.org/10.1061/(asce)0733-9372(2005)131:8(1203>).

[7] S.H. Liang, K.F. Chen, C.S. Wu, Y.H. Lin, C.M. Kao, Development of KMnO4-releasing composites for in situ chemical oxidation of TCE-contaminated groundwater, Water Research 54 (2014) 149-158. <https://doi.org/https://doi.org/10.1016/j.watres.2014.01.068>.
